# The novel microtubule-interfering agent TZT-1027 enhances the anticancer effect of radiation *in vitro* and *in vivo*

**DOI:** 10.1038/sj.bjc.6603769

**Published:** 2007-05-01

**Authors:** Y Akashi, I Okamoto, M Suzuki, K Tamura, T Iwasa, S Hisada, T Satoh, K Nakagawa, K Ono, M Fukuoka

**Affiliations:** 1 Department of Medical Oncology, Kinki University School of Medicine, 377-2 Ohno-higashi, Osaka-Sayama, Osaka 589-8511, Japan; 2 Radiation Oncology Research Laboratory, Research Reactor Institute, Kyoto University, 2-1010 Asashiro-nishi, Kumatori-cho, Sennan-gun, Osaka 590-0494, Japan; 3 Department of Medical Oncology, Kinki University School of Medicine, Nara Hospital, 1248-1 Otodacho, Ikoma, Nara 630-0293, Japan; 4 Asuka Pharmaceutical Co. Ltd, 1604 Shimosakunobe, Takatu-ku, Kawasaki 213-8522, Japan

**Keywords:** TZT-1027, radiosensitisation, microtubule, mitotic arrest, apoptosis, antivascular effect

## Abstract

TZT-1027 is a novel anticancer agent that inhibits microtubule polymerisation and manifests potent antitumour activity in preclinical models. We have examined the effect of TZT-1027 on cell cycle progression as well as the anticancer activity of this drug both *in vitro* and *in vivo*. With the use of tsFT210 cells, which express a temperature-sensitive mutant of Cdc2, we found that TZT-1027 arrests cell cycle progression in mitosis, the phase of the cell cycle most sensitive to radiation. A clonogenic assay indeed revealed that TZT-1027 increased the sensitivity of H460 cells to *γ*-radiation, with a dose enhancement factor of 1.2. Furthermore, TZT-1027 increased the radiosensitivity of H460 and A549 cells in nude mice, as revealed by a marked delay in tumour growth and an enhancement factor of 3.0 and 2.2, respectively. TZT-1027 also potentiated the induction of apoptosis in H460 cells by radiation both *in vitro* and *in vivo*. Histological evaluation of H460 tumours revealed that TZT-1027 induced morphological damage to the vascular endothelium followed by extensive central tumour necrosis. Our results thus suggest that TZT-1027 enhances the antitumour effect of ionising radiation, and that this action is attributable in part to potentiation of apoptosis induction and to an antivascular effect. Combined treatment with TZT-1027 and radiation therefore warrants investigation in clinical trials as a potential anticancer strategy.

The combination of modalities of cancer treatment offers improvements in the survival of cancer patients compared with individual therapeutic approaches. Such therapeutic benefit has been achieved with combinations of chemo- and radiotherapy in a variety of cancers. The cytotoxicity of most chemotherapeutic agents as well as that of radiation is highly dependent on the phase of the cell cycle. Although various types of anticancer drugs are able to arrest cells at specific cell cycle checkpoints, the ability of antimicrotubule agents to block cell cycle progression in G_2_-M phase is the biological basis for combination of these agents with radiation (Pawlik and Keyomarsi, 2004). Microtubule-interfering agents have been shown to increase the radiosensitivity of tumour cells in preclinical and clinical studies (Liebmann *et al*, 1994; Choy *et al*, 1995; Edelstein *et al*, 1996; Vokes *et al*, 1996; Kim *et al*, 2001, 2003, 2003; Hofstetter *et al*, 2005; Simoens *et al*, 2006).

TZT-1027 (Soblidotin), a novel microtubule-interfering agent synthesised from dolastatin 10 (Figure 1), exhibits greater antitumour activities and a reduced toxicity compared with its parent compound (Miyazaki *et al*, 1995). TZT-1027 inhibits microtubule assembly by binding to tubulin (Kobayashi *et al*, 1997; Natsume *et al*, 2000). *In vitro*, it inhibits the growth of various human cancer cells at low concentrations (Watanabe *et al*, 2000). *In vivo*, TZT-1027 also manifests a broad spectrum of activity against various murine tumours as well as human tumour xenografts, without inducing a pronounced reduction in body weight (Kobayashi *et al*, 1997; Watanabe *et al*, 2000, 2006a; Natsume *et al*, 2003, 2006). Furthermore, the drug exhibited a potent antivascular effect on existing vasculature in an advanced-stage tumour model (Otani *et al*, 2000). TZT-1027 is currently undergoing clinical evaluation, with a reduction in tumour size and disease stabilisation having been observed in a subset of patients (Schoffski *et al*, 2004; de Jonge *et al*, 2005; Greystoke *et al*, 2006; Tamura *et al*, 2007).

Despite its demonstrated efficacy against solid tumours, the effects of TZT-1027 in combination with radiation have not been examined. As an initial step in determining the antitumour activity of TZT-1027 in combination with radiation, we investigated the effect of this agent on cell cycle progression in synchronised tsFT210 cells (Osada *et al*, 1997), which harbour a temperature-sensitive mutant of Cdc2. We found that TZT-1027 induces arrest of cells in mitosis, the phase of the cell cycle most sensitive to radiation. We then studied the radiosensitising properties of TZT-1027 *in vitro* and *in vivo* with a human lung cancer model and elucidated the mechanism of radiosensitisation by this agent.

## MATERIALS AND METHODS

### Cell lines and reagents

**Figure 1:**
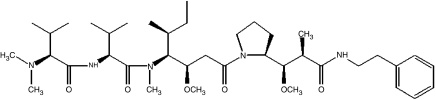
Chemical structure of TZT-1027.

tsFT210 mouse mammary carcinoma cells, which express a temperature-sensitive mutant of Cdc2, were kindly provided by H Kakeya (Antibiotics Laboratory, Discovery Research Institute, RIKEN, Saitama, Japan) and were maintained under a humidified atmosphere of 5% CO_2_ in air at 32.0°C in RPMI 1640 (Sigma, St Louis, MO, USA) supplemented with 10% foetal bovine serum and 1% penicillin-streptomycin. H460 human lung large cell carcinoma and A549 human lung adenocarcinoma cells were obtained from American Type Culture Collection (Manassas, VA, USA) and were maintained as for tsFT210 cells with the exception that the culture temperature was 37°C. TZT-1027 (Figure 1) was provided by Daiichi Pharmaceutical Co. Ltd (Tokyo, Japan). Nocodazole and roscovitine were obtained from Sigma.

### Cell cycle analysis

Cells were harvested, washed with phosphate-buffered saline (PBS), fixed with 70% methanol, washed again with PBS, and stained with propidium iodide (0.05 mg ml^−1^) in a solution containing 0.1% Triton X-100, 0.1 mM EDTA, and RNase A (0.05 mg ml^−1^). The stained cells (∼1 × 10^6^) were then analysed for DNA content with a flow cytometer (FACScalibur; Becton Dickinson, San Jose, CA, USA).

### Measurement of mitotic index and apoptotic cells

Cells were harvested, washed with PBS, fixed with methanol : acetic acid (3 : 1, v/v), washed again with PBS, and stained with 4′,6-diamidino-2-phenylindole (DAPI) (0.5 *μ*g ml^−1^). The stained cells (∼1 × 10^6^) were observed with a fluorescence microscope (IX71; Olympus, Tokyo, Japan). To determine the proportion of mitotic or apoptotic cells, we scored at least 300 cells in each of at least three randomly selected microscopic fields for each of three slides per sample. Cells with condensed chromosomes and no obvious nuclear membrane were regarded as mitotic cells, and the mitotic index was calculated as the percentage of mitotic cells among total viable cells. Cells with fragmented and uniformly condensed nuclei were regarded as apoptotic cells.

### Clonogenic assay

Exponentially growing H460 cells in 25-cm^2^ flasks were harvested by exposure to trypsin and counted. They were diluted serially to appropriate densities and plated in triplicate in 25-cm^2^ flasks containing 10 ml of medium. The cells were treated with 1 nM TZT-1027 or vehicle (dimethyl sulfoxide, or DMSO; final concentration, 0.1%) for 24 h and then exposed to various doses of *γ*-radiation with a ^60^Co irradiator at a rate of ∼0.82 Gy min^−1^ and at room temperature. The cells were then washed with PBS, cultured in drug-free medium for 10–14 days, fixed with methanol : acetic acid (10 : 1, v/v), and stained with crystal violet. Colonies containing >50 cells were counted. The surviving fraction was calculated as: (mean number of colonies)/(number of inoculated cells × plating efficiency). Plating efficiency was defined as the mean number of colonies divided by the number of inoculated cells for nonirradiated controls. The surviving fraction for combined treatment was corrected by that for TZT-1027 treatment alone. The dose enhancement factor (DEF) was calculated as the dose (Gy) of radiation that yielded a surviving fraction of 0.1 for vehicle-treated cells divided by that for TZT-1027-treated cells (after correction for drug toxicity).

### *In vivo* antitumour activity of TZT-1027 with or without radiation

All animal studies were performed in accordance with the Recommendations for Handling of Laboratory Animals for Biomedical Research, compiled by the Committee on Safety and Ethical Handling Regulations for Laboratory Animal Experiments, Kyoto University. The ethical guidelines followed meet the requirements of the UKCCCR guidelines (Workman *et al*, 1998). Tumour cells (2 × 10^6^) were injected subcutaneously into the right hind leg of 7-week-old female athymic nude mice. Tumour volume was determined from caliper measurement of tumour length (*L*) and width (*W*) according to the formula *LW*^2^/2. Treatment was initiated when tumours in each group achieved an average volume of ∼200–250 mm^3^. Treatment groups consisted of control, TZT-1027 alone, radiation alone, and the combination of TZT-1027 and radiation. Each treatment group contained six to eight mice. TZT-1027 was administered intravenously in a single dose of 0.5 mg kg^−1^ of body weight; mice in the control and radiation-alone groups were injected with vehicle (physiological saline). Tumours in the leg were exposed to 10 Gy of *γ*-radiation with a ^60^Co irradiator at a rate of ∼0.32 Gy min^−1^ immediately after drug treatment. Growth delay (GD) was calculated as the time for treated tumours to achieve an average volume of 500 mm^3^ minus the time for control tumours to reach 500 mm^3^. The enhancement factor was then determined as: (GD_combination_–GD_TZT−1027_)/(GD_radiation_).

### TUNEL staining

Mice were killed 14 days after treatment initiation and the tumours were removed and preserved in 10% paraformaldehyde. Apoptosis in tumour sections was determined by the terminal deoxynucleotidyl transferase-mediated dUTP-biotin nick-end labelling (TUNEL) assay with the use of an apoptosis detection kit (Chemicon, Temecula, CA, USA). The number of apoptotic cells was counted in 10 separate microscopic fields (× 100) for three sections of each tumour of each group.

### Histological analysis

A single dose of TZT-1027 (2.0 mg kg^−1^) or vehicle (physiological saline) was administered intravenously to mice when H460 tumours had achieved a volume of ∼400 to 600 mm^3^. Tumour tissue was extirpated 4 or 24 h after drug administration, and half of the tissue was fixed in 10% buffered formalin, embedded in paraffin, sectioned, and stained with hematoxylin-eosin. The other half of the tumour tissue was fixed for 12–48 h in zinc fixative (BD Biosciences, San Jose, CA, USA), embedded in paraffin, sectioned, and immunostained for CD31. Endogenous peroxidase activity was blocked by incubation of the latter sections for 20 min with 0.3% H_2_O_2_ in methanol, and nonspecific sites were blocked with antibody diluent (Dako Japan, Kyoto, Japan). Sections were then incubated overnight at 4°C with a 1 : 50 dilution of a rat monoclonal antibody to mouse CD31 (BD Biosciences), washed with PBS, and processed with a Histfine Simple Stain PO (M) kit (Nichirei, Tokyo, Japan) for detection of immune complexes. Sections were counterstained with Mayer's hematoxylin, covered with a coverslip with the use of a permanent mounting medium, and examined with a light microscope (CX41; Olympus, Tokyo, Japan).

### Statistical analysis

Data are presented as means±s.d. or s.e. and were compared by the unpaired Student's *t*-test. A *P* value of <0.05 was considered statistically significant.

## RESULTS

### Induction of cell cycle arrest at M phase but not at G_1_-S in tsFT210 cells by TZT-1027

**Figure 2:**
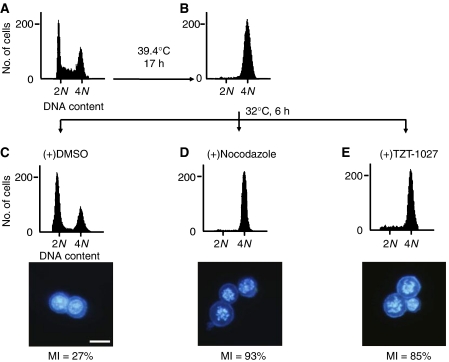
Inhibition of tsFT210 cell cycle progression through G_2_-M by TZT-1027. Cells were cultured at the permissive temperature of 32.0°C (**A**) and then incubated for 17 h at the nonpermissive temperature of 39.4°C (**B**). They were subsequently released from G_2_ arrest by incubation at 32.0°C for 6 h in the presence of DMSO (**C**), 1 *μ*M nocodazole (**D**), or 2 nM TZT-1027 (**E**). At each stage of the protocol, cells were analysed for DNA content by staining with propidium iodide and flow cytometry. The 2N and 4N peaks indicate cells in G_0_-G_1_ and G_2_-M phases of the cell cycle, respectively. The cells were also stained with DAPI and examined by fluorescence microscopy after treatment with DMSO, nocodazole, or TZT-1027 (lower panels), and the mitotic index (MI) was determined; scale bar, 20 *μ*m. Data are representative of at least three independent experiments.

To examine the effect of TZT-1027 on cell cycle progression, we performed flow cytometric analysis of tsFT210 cells, which express a temperature-sensitive mutant of Cdc2. These mammary carcinoma cells exhibit a normal cell cycle distribution when incubated at the permissive temperature of 32.0°C, but they arrest at G_2_ phase as a result of Cdc2 inactivation when incubated at the nonpermissive temperature of 39.4°C (Figure 2A and B). We synchronised tsFT210 cells at G_2_ phase by incubation at 39.4°C for 17 h and then cultured them at 32.0°C for 6 h in the presence of nocodazole (an inhibitor of microtubule polymerisation), TZT-1027, or vehicle (DMSO). In the presence of vehicle alone, the number of cells in G_2_ phase decreased markedly and there was a corresponding increase in the number of cells in G_1_ phase, indicative of re-entry of cells into the cell cycle (Figure 2C). In contrast, treatment with nocodazole or TZT-1027 prevented the cells from passing through G_2_-M phase (Figure 2D and E). Given that flow cytometric analysis did not distinguish between cells in M phase and those in G_2_ phase, we determined the mitotic index of cells by DAPI staining and fluorescence microscopy. Most of the cells released from temperature-induced arrest in the presence of nocodazole manifested condensed chromosomes without a nuclear membrane, yielding a mitotic index of 93%; most of the cells had thus arrested in mitosis (Figure 2D). Most of the cells released from temperature-induced arrest in the presence of TZT-1027 showed similar mitotic figures, yielding a mitotic index of 85% (Figure 2E) and demonstrating that TZT-1027 also inhibits cell cycle progression at mitosis.

**Figure 3:**
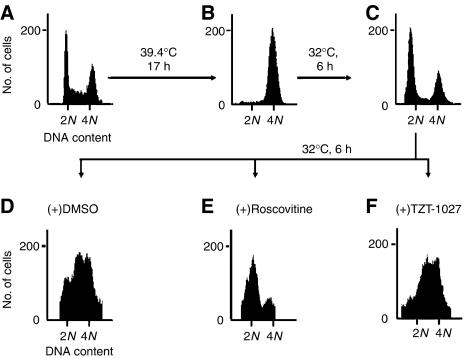
Lack of effect of TZT-1027 on tsFT210 cell cycle progression through G_1_-S. Exponentially growing tsFT210 cells (**A**) were arrested in G_2_ phase by incubation for 17 h at 39.4°C (**B**). The cells were incubated at 32.0°C first for 6 h to allow progression to G_1_ phase (**C**) and then for an additional 6 h in the presence of DMSO (**D**), 50 *μ*M roscovitine (**E**), or 2 nM TZT-1027 (**F**). At each stage of the protocol, cells were analysed for DNA content by flow cytometry. Data are representative of at least three independent experiments.

We next examined whether TZT-1027 affects the G_1_-S transition. We arrested tsFT210 cells at G_2_ phase by incubation at 39.4°C, released the cells into G_1_ phase by shifting to the permissive temperature for 6 h, and then incubated them for an additional 6 h in the presence of roscovitine (an inhibitor of CDK2 that prevents cell cycle progression at G_1_ phase), TZT-1027, or vehicle (Figure 3). The cells incubated with vehicle passed through G_1_ phase and yielded a broad S-phase peak (Figure 3D), whereas those treated with roscovitine did not pass through G_1_ phase (Figure 3E). In contrast, TZT-1027 had no effect on passage of the synchronised tsFT210 cells through the G_1_-S transition (Figure 3F). Together, these results indicate that the effect of TZT-1027 on cell cycle progression is specific to M phase.

### Induction of cell cycle arrest at M phase in asynchronous H460 cells by TZT-1027

**Figure 4:**
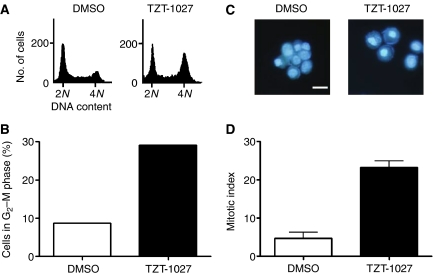
Induction of cell cycle arrest at M phase in H460 cells by TZT-1027. H460 cells were incubated in the presence of 1 nM TZT-1027 or vehicle (DMSO) for 24 h, after which DNA content was measured by flow cytometry (**A**) and the fraction of cells in G_2_-M phase was determined (**B**). The cells were also stained with DAPI and examined by fluorescence microscopy (**C**) and the mitotic index was determined (**D**). Data in (**A**) through (**C**) are representative of at least three independent experiments; data in (**D**) are means±s.d. of values from three independent experiments. Scale bar in (**C**), 20 *μ*m.

We next examined whether TZT-1027 induced mitotic arrest in asynchronous H460 human non-small cell lung cancer cells. Flow cytometric analysis revealed that treatment of H460 cells with TZT-1027 for 24 h induced a threefold increase in the proportion of cells with a DNA content of 4N compared with that apparent for vehicle-treated cells (29.1 *vs* 8.7%) (Figure 4A and B). Furthermore, DAPI staining revealed that TZT-1027 induced a significant increase in the mitotic index of H460 cells compared with that for the control cells (23.3 *vs* 4.6%) (Figure 4C and D), indicating that most of the TZT-1027-treated cells with a DNA content of 4N were arrested in M phase rather than in G_2_ phase. These observations thus showed that TZT-1027 also induced mitotic arrest in asynchronous H460 cells.

### Radiosensitisation of H460 cells by TZT-1027 *in vitro*

**Figure 5:**
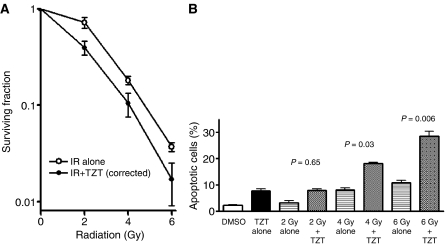
Sensitisation of H460 cells to *γ*-radiation by TZT-1027 *in vitro*. (**A**) Clonogenic assay. Cells were incubated with 1 nM TZT-1027 or vehicle (DMSO) for 24 h, exposed to the indicated doses of *γ*-radiation, and then incubated in drug-free medium for 10–14 days for determination of colony-forming ability. Survival curves were generated after correction of colony formation observed for combined treatment with ionising radiation (IR) and TZT-1027 by that apparent for treatment with TZT-1027 alone. (**B**) Assay of apoptosis. Cells were incubated with 1 nM TZT-1027 or vehicle (DMSO) for 24 h, exposed to various doses (0, 2, 4, or 6 Gy) of *γ*-radiation, and then incubated for 24 h in drug-free medium. Cells were then fixed and stained with DAPI for determination of the proportion of apoptotic cells by fluorescence microscopy. Data in (**A**) and (**B**) are means±s.d. of values from three independent experiments. *P* values in (**B**) are for comparison of the value for combined treatment with TZT-1027 and radiation *vs* the sum of the corresponding values for each treatment alone, after correction of all data by the control (DMSO) value.

Cells in M phase are more sensitive to radiation than are those in other phases of the cell cycle. Given that exposure of H460 cells to TZT-1027-induced mitotic arrest, we next examined whether this agent might sensitise H460 cells to *γ*-radiation with the use of a clonogenic assay. H460 cells were incubated for 24 h with 1 nM TZT-1027 or vehicle (DMSO) and then exposed to various doses (0, 2, 4, or 6 Gy) of *γ*-radiation. The cells were then allowed to form colonies in drug-free medium for 10–14 days. Survival curves revealed that TZT-1027 increased the radiosensitivity of H460 cells, with a DEF of 1.2 (Figure 5A).

To determine whether radiosensitisation by TZT-1027 was reflected by an increase in the proportion of apoptotic cells, we exposed H460 cells to 1 nM TZT-1027 or vehicle for 24 h, treated the cells with various doses (0, 2, 4, or 6 Gy) of radiation, and then incubated them in drug-free medium for an additional 24 h before quantification of apoptosis. Combined treatment with TZT-1027 and 4 or 6 Gy of radiation resulted in a significant increase in the number of apoptotic cells compared with the sum of the values for treatment with drug alone or radiation alone (Figure 5B). TZT-1027 thus promoted radiation-induced apoptosis in H460 cells.

### Radiosensitisation of H460 cells and A549 cells by TZT-1027 *in vivo*

**Figure 6:**
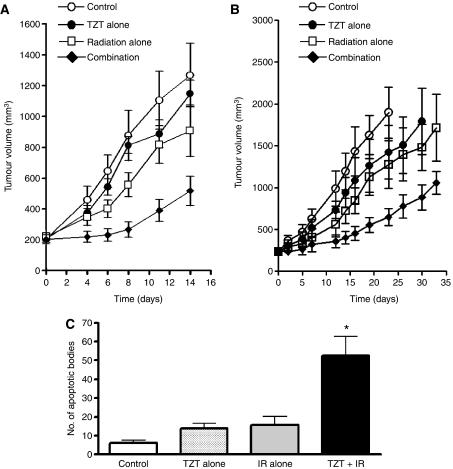
Sensitisation of H460 and A549 cells to *γ*-radiation by TZT-1027 *in vivo*. (**A** and **B**) Evaluation of tumour growth. Nude mice with H460 (**A**) or A549 (**B**) tumour xenografts (∼200 to 250 mm^3^) were treated with a single intravenous dose of TZT-1027 (0.5 mg kg^−1^), a single dose of *γ*-radiation (10 Gy), or neither (control) or both modalities, and tumour volume was determined at the indicated times thereafter. Data are means±s.e. for six to eight mice per group. (**C**) Quantification of apoptotic cells in H460 tumour sections by TUNEL staining 14 days after the initiation of treatment as in (**A**). Data are means±s.d. ^*^P<0.05 *vs* mice treated with TZT-1027 alone or radiation alone.

To determine whether the TZT-1027-induced increase in the radiosensitivity of tumour cells observed *in vitro* might also be apparent *in vivo*, we injected H460 cells or A549 human lung adenocarcinoma cells into nude mice in order to elicit the formation of solid tumours. The mice were then treated with TZT-1027, radiation, or both modalities. Treatment with TZT-1027 alone (single dose of 0.5 mg kg^−1^) or with radiation alone (single dose of 10 Gy) resulted in relatively small inhibitory effects on tumour growth, whereas combined treatment with both TZT-1027 and radiation exerted a markedly greater inhibitory effect (Figure 6A and B). The tumour GDs induced by treatment with TZT-1027 alone, radiation alone, or both TZT-1027 and radiation were 1.0, 2.6, and 8.8 days, respectively, for H460 cells and 1.4, 4.9, and 12.4 days, respectively, for A549 cells (Table 1). The enhancement factor for the effect of TZT-1027 on the efficacy of radiation was 3.0 for H460 cells and 2.2 for A549 cells, revealing the effect to be greater than additive. No pronounced tissue damage or toxicities such as diarrhoea or weight loss of >10% were observed in mice in any of the four treatment groups (Table 2).

We examined the effects of the treatment protocols on apoptosis in H460 tumours by TUNEL staining of tumour sections. Quantification of the number of apoptotic cells revealed that the combined treatment with radiation and TZT-1027 induced a significant increase in this parameter compared with treatment with radiation or TZT-1027 alone (Figure 6C).

### Histological appearance of H460 tumours after administration of TZT-1027

**Figure 7:**
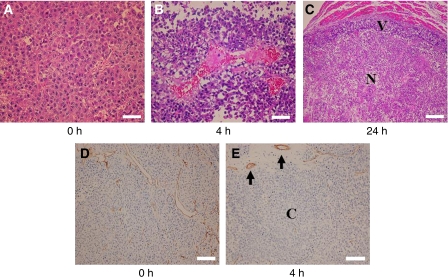
Histological analysis of H460 tumours after treatment with TZT-1027. Mice bearing H460 tumour xenografts were treated with a single dose of TZT-1027 (2.0 mg kg^−1^), and the tumours were excised at various times thereafter and either stained with hematoxylin-eosin (**A**–**C**) or immunostained for CD31 (**D** and **E**). (**A** and **D**) Control sections of an untreated tumour showing normal capillaries with an intact endothelium and viable tumour cells. (**B** and **E**) Sections of a tumour removed 4 h after administration of TZT-1027. Vascular congestion, with pink deposits of fibrin, and loss of endothelial cells as well as diffuse tumour cell degeneration are apparent in (**b**). Dark immunostaining of intact endothelium (arrows) is apparent in surrounding normal connective tissue, whereas little staining of endothelial cells was observed in the core (**C**) of the tumour (**E**). (**C**) Section of a tumour removed 24 h after TZT-1027 administration, showing extensive central necrosis (N) and a rim of viable cells (V). Scale bars: 50 *μ*m (**A** and **B**), 100 *μ*m (**C**), and 200 *μ*m (**D** and **E**).

Finally, we examined whether an effect of TZT-1027 on tumour vasculature might contribute to the antitumour activity of this drug *in vivo*. Mice harbouring H460 tumours were injected with TZT-1027, and the tumours were excised 4 or 24 h thereafter and examined by hematoxylin-eosin staining (Figure 7A–C) or by immunostaining for the endothelial cell marker CD31 (Figure 7D and E). Tumour capillaries appeared congested, with thrombus formation, and showed a loss of endothelial cells 4 h after administration of TZT-1027 (Figure 7B and E), whereas vessels within viable areas of control tumours were generally not congested and showed an intact normal endothelium (Figure 7A and D). The effects of TZT-1027 on the tumour vasculature appeared selective, given that neither loss of CD31 staining nor vessel congestion was apparent in the vasculature of surrounding normal tissue after drug treatment (Figure 7E). Extensive necrosis was apparent at the tumour core, with a characteristic thin rim of viable tumour cells remaining at the periphery, 24 h after TZT-1027 administration (Figure 7C). These results were thus indicative of a characteristic antivascular effect of TZT-1027 in the H460 tumour model.

## DISCUSSION

TZT-1027 is a novel antitumour agent that inhibits microtubule polymerisation and exhibits potent antitumour activity in preclinical models (Miyazaki *et al*, 1995; Kobayashi *et al*, 1997; Natsume *et al*, 2000, 2003, 2006; Otani *et al*, 2000; Watanabe *et al*, 2000, 2006a). We investigated the effect of TZT-1027 on cell cycle progression with the use of tsFT210 cells, which can be synchronised in G_2_ phase by incubation at 39.4°C and consequent inactivation of Cdc2 (Osada *et al*, 1997; Tamura *et al*, 1999). The use of these cells allows cell synchronisation without the need for agents that prevent DNA synthesis (such as hydroxyurea or thymidine) or that inhibit formation of the mitotic spindle (such as nocodazole). Although such agents halt cell cycle progression in specific phases of the cycle, they are also toxic and kill a proportion of the treated cells. The tsFT210 cell system is thus suited to sensitive analysis of the effects of new compounds on cell cycle progression without loss of cell viability. We have now shown that tsFT210 cells released from G_2_ arrest by incubation at 32.0°C failed to pass through M phase in the presence of TZT-1027. Although previous flow cytometric analysis of exponentially growing tumour cells revealed that TZT-1027 induced a marked increase in the proportion of cells in G_2_-M (Watanabe *et al*, 2000), it was uncertain whether the drug arrested cell cycle progression in G_2_ or in mitosis. Our morphological data now indicate that, similar to the effect of nocodazole, TZT-1027 arrested tsFT210 cells in M phase rather than in G_2_, consistent with the mode of action of this new compound. Given that microtubules contribute to various cellular functions in addition to cell division, including intracellular transport and signal transduction (Mollinedo and Gajate, 2003), TZT-1027 might also be expected to affect tumour cells in interphase. With the use of synchronised tsFT210 cells, however, we found that TZT-1027 had no effect on progression of cells through the G_1_–S transition of the cell cycle. The effect of TZT-1027 on cell cycle progression thus appears to be specific to M phase.

Given that cells are most sensitive to radiation during mitosis (Sinclair and Morton, 1966; Sinclair, 1968; Pawlik and Keyomarsi, 2004), we next investigated the possible interaction between TZT-1027 and ionising radiation in human lung cancer cell lines. We found that TZT-1027 increased the sensitivity of H460 cells to *γ*-radiation *in vitro*. The proportion of H460 cells in mitotic phase at the time of irradiation was increased by TZT-1027 treatment, consistent with the notion that this effect contributes to the observed radiosensitisation induced by this drug. TZT-1027 was previously shown to induce apoptosis in several tumour cell lines (Watanabe *et al*, 2000). Although the relation between apoptosis and radiosensitivity is controversial (Lawrence *et al*, 2001; Pawlik and Keyomarsi, 2004), we showed that treatment of H460 cells with TZT-1027 before irradiation induced a marked increase in the proportion of apoptotic cells compared with that apparent with radiation alone. These results thus suggested that potentiation of apoptosis contributed to radiosensitisation by TZT-1027.

Combined treatment with radiation and a single administration of TZT-1027 also inhibited the growth of tumours formed by H460 or A549 cells *in vivo* to a greater extent than did either treatment alone. Tumour microenvironmental factors, such as the vascular supply, are important determinants of sensitivity to radiation therapy *in vivo*. The ability of microtubule-targeting agents to induce a rapid shutdown of the existing tumour vasculature has been recognised by their designation as vascular-targeting agents (VTAs) (Jordan and Wilson, 2004). Treatment with VTAs such as ZD6126 and combretastatin A-4-P typically results in the destruction of large areas of a tumour, with surviving cells remaining only at the tumour periphery (Dark *et al*, 1997; Blakey *et al*, 2002). These peripheral viable tumour cells presumably derive their nutritional support from nearby normal blood vessels that are not responsive to VTA treatment (Li *et al*, 1998; Siemann and Rojiani, 2002). Such support together with a rapid upregulation of angiogenic factors such as vascular endothelial growth factor may directly facilitate the growth and expansion of the remaining tumour cells (Wachsberger *et al*, 2003; Thorpe, 2004). Given that these residual tumour cells are likely well oxygenated (Wachsberger *et al*, 2003), they are an ideal target for radiation therapy. Several studies have recently shown that treatment with VTAs enhances the therapeutic effect of radiotherapy (Li *et al*, 1998; Siemann and Rojiani, 2002, 2005; Horsman and Murata, 2003; Masunaga *et al*, 2004), consistent with the idea that the components of such combination therapy act in a complementary manner, with VTAs attacking the poorly oxygenated cell population in the central region of tumours and radiation killing the well-oxygenated proliferating cells at the tumour periphery (Li *et al*, 1998; Siemann and Rojiani, 2002; Wachsberger *et al*, 2003). TZT-1027 was previously shown to increase vascular permeability and to induce a decrease in tumour blood flow followed by a marked increase in tissue necrosis in the central region of tumour xenografts (Otani *et al*, 2000; Watanabe *et al*, 2006b). We have now shown that TZT-1027 treatment resulted in congestion and occlusion of tumour blood vessels followed by extensive necrosis of the tumour core, with only a thin rim of viable tumour cells remaining, in the H460 tumour model, suggesting that TZT-1027 acts as a VTA. The action of TZT-1027 as a VTA might thus contribute to the radiosensitising effect observed *in vivo* in the present study.

The clinical use of microtubule-interfering agents such as taxanes in combination with radiation has been successful in improving local tumour control. However, taxanes are often of limited efficacy because of the development of cellular resistance such as that mediated by P-glycoprotein-dependent drug efflux (Goodin *et al*, 2004). The action of TZT-1027 has been suggested to be less affected by multidrug resistance factors, including overexpression of P-glycoprotein, than that of other tubulin inhibitors (Watanabe *et al*, 2006a), suggesting that TZT-1027 may be effective in the treatment of taxane-refractory tumours. Further investigations are thus warranted to examine the combined effects of TZT-1027 and ionising radiation on drug-resistant tumour cells. Whether TZT-1027 enhances the tumour response to clinically relevant fractionated doses of radiation such as 2 Gy per fraction also warrants further study.

In conclusion, we have found that the inhibitory effect of TZT-1027 on cell cycle progression is highly specific to M phase. Moreover, TZT-1027 enhanced the effects of radiation on human cancer cells both *in vitro* and in animal models *in vivo*. These preclinical results provide a rationale for future clinical investigations of the therapeutic efficacy of TZT-1027 in combination with radiotherapy.

## Figures and Tables

**Table 1:** Tumour growth delay value

	**H460**		**A549**	
**Treatment**	**Days** a	**GD** b	**Days**	**GD**
Control	4.5		5.5	
TZT-1027 alone	5.5	1	6.9	1.4
Radiation alone	7.1	2.6	10.4	4.9
TZT-1027 + Radiation	13.3	8.8	17.9	12.4
Enhancement factor	3		2.2	

a Days, the period needed for the sizes of xenografts in each group to reach 500 mm^3^;

b GD, the additional periods needed for the sizes of xenografts in each group to reach 500 mm^3^ in addition to the period needed for controls to reach 500 mm^3^.

**Table 2:** Body weight loss

	**% of B.W.L** a	
	**H460**	**A549**
Control	3.6	1.2
TZT-1027 alone	9.9	5.2
Radiation alone	9.7	5.5
TZT-1027+Radiation	8.7	9.9

a % of B.W.L, relative body weight loss 7 days after the initiation of the treatment.
